# Classically and alternatively activated bone marrow derived macrophages differ in cytoskeletal functions and migration towards specific CNS cell types

**DOI:** 10.1186/1742-2094-8-58

**Published:** 2011-05-26

**Authors:** Elly JF Vereyken, Priscilla DAM Heijnen, Wia Baron, Elga HE de Vries, Christine D Dijkstra, Charlotte E Teunissen

**Affiliations:** 1 Department of Molecular Cell Biology and Immunology, VU University Medical Center, Amsterdam, the Netherlands; 2 University Medical Center Groningen University of Groningen, Dept. Cell Biology, Section Membrane Cell Biology, Groningen, the Netherlands; 3 Department of Clinical chemistry, VU University Medical Center, Amsterdam, the Netherlands

**Keywords:** migration, classically activated macrophages, alternatively activated macrophages, central nervous system, neurons

## Abstract

**Background:**

Macrophages play an important role in neuroinflammatory diseases such as multiple sclerosis (MS) and spinal cord injury (SCI), being involved in both damage and repair. The divergent effects of macrophages might be explained by their different activation status: classically activated (CA/M1), pro-inflammatory, macrophages and alternatively activated (AA/M2), growth promoting, macrophages. Little is known about the effect of macrophages with these phenotypes in the central nervous system (CNS) and how they influence pathogenesis. The aim of this study was therefore to determine the characteristics of these phenotypically different macrophages in the context of the CNS in an *in vitro *setting.

**Results:**

Here we show that bone marrow derived CA and AA macrophages have a distinct migratory capacity towards medium conditioned by various cell types of the CNS. AA macrophages were preferentially attracted by the low weight (< 10 kD) fraction of neuronal conditioned medium, while CA macrophages were attracted in higher numbers by astrocyte- and oligodendrocyte conditioned medium. Intrinsic motility was twice as high in AA macrophages compared to CA macrophages. The adhesion to extracellular matrix molecules (ECM) was significantly enhanced in CA macrophages compared to control and AA macrophages. The actin cytoskeleton was differentially organized between CA and AA macrophages, possibly due to greater activity of the GTPases RhoA and Rac in CA macrophages. Phagocytosis of myelin and neuronal fragments was increased in CA macrophages compared to AA macrophages. The increase in myelin phagocytosis was associated with higher expression of CR3/MAC-1 in CA macrophages.

**Conclusion:**

In conclusion, since AA macrophages are more motile and are attracted by NCM, they are prone to migrate towards neurons in the CNS. CA macrophages have a lower motility and a stronger adhesion to ECM. In neuroinflammatory diseases the restricted migration and motility of CA macrophages might limit lesion size due to bystander damage.

## Background

Macrophages are phagocytic cells that play an essential role in both innate and acquired immunity. Macrophages are not a homogeneous cell population, since they are highly plastic cells that are able to respond to a variety of environmental cues by changing their phenotype and physiology [1,2]. The two phenotypes that are considered to be the most extreme are classically activated (CA/M1) pro-inflammatory macrophages and alternatively activated (AA/M2) or growth promoting macrophages. In tissues, the micro-environment of the macrophages is thought to determine the phenotype [2]. *In vitro*, cytokines and other stimuli induce these activation phenotypes. CA macrophages are induced by interferon-gamma (IFN-γ) and lipopolysaccharide (LPS). The induction of the AA phenotype is not straightforward, due to the fact that a range of stimuli, such as IL-4/IL-13, IL-10, immunocomplexes and glucocorticoids, are reported to induce alternative activation in macrophages [2]. Consequently, a wide variety of phenotypical and functional characteristics have been attributed to alternatively activated macrophages. The most common and widely studied way to generate AA macrophages is by exposure to interleukin (IL)-4/IL-13 [1,3]. Notably, IL-4 stimulated AA and CA macrophages have distinct functions in tissue repair and inflammation. The CA macrophages produce nitric oxide (NO) and reactive oxygen species (ROS) making them cytotoxic [1,4]. Furthermore, they secrete high amounts of pro-inflammatory cytokines, for example IL-12, that promote inflammation. Conversely, AA macrophages produce anti-inflammatory cytokines, such as IL-10 [1,2], thereby reducing inflammation. AA macrophages have a higher angiogenic potential compared to CA macrophages [5]. They produce growth factors, such as platelet derived growth factor (PDGF), vascular endothelial growth factor (VEGF) and fibroblast growth factor (FGF) [5-7]. In mice, AA macrophages express high levels of arginase. Due to activation of arginase, arginine is converted to ornithine, a precursor for collagen, which contributes to the production of extracellular matrix molecules (ECM) [8-11] and promotes cell growth [10,12]. Together these features determine the wound healing and growth promoting phenotype of AA macrophages [1,2].

Macrophages can play crucial roles during neuroinflammatory diseases, such as spinal cord injury (SCI) and multiple sclerosis (MS). MS is a chronic inflammatory disease of the central nervous system (CNS). Major neuropathological hallmarks of MS are inflammatory demyelinating lesions associated with perivascular infiltrates containing macrophages and lymphocytes [13,14]. It is widely accepted that macrophages play an important role during MS pathogenesis and both detrimental and beneficial effects of macrophages have been observed during MS and experimental autoimmune encephalomyelitis (EAE), an animal model for MS. Activated macrophages secrete many products that could contribute to axonal and oligodendrocyte damage seen in MS lesions, such as matrix metalloproteinases (MMPs) [15], NO and glutamate, [16-22]. The elimination of infiltrating macrophages by clodronate liposomes suppressed clinical signs of EAE [23,24]. A correlation was found between location and amount of axonal damage and loss and cellular infiltrates containing macrophages [25-27]. These studies suggest that macrophages play a detrimental role, but beneficial effects have been reported for macrophages as well. Phagocytosis of myelin debris is important for axonal repair/regrowth and remyelination, since myelin components are known to be growth inhibiting [28,29]. Moreover, activated macrophages/microglia are found to be sources of neurotrophins and growth factors, such as brain derived neurotrophic factor (BDNF) in MS lesions [30,31] and elimination of infiltrating macrophages reduced remyelination in demyelinating models [32].

These divergent effects of macrophages have also been observed during SCI. As with MS, depletion of infiltrating macrophages was beneficial, indicating that macrophages are detrimental. Elimination of infiltrating macrophages was observed to increase axonal repair and functional outcome [33-35]. However, macrophages can also create a growth-permissive environment in which axonal regeneration can take place during SCI [36-38]. Kigerl *et al. *observed that CA macrophages predominate in the SCI lesion, while the AA macrophage response is only transient in lesions, possibly explaining limited repair [39].

The divergent effects of macrophages in neuroinflammatory diseases might be due to the different activation states that macrophages can acquire. Little research has been done on AA and CA macrophages and their functions in the CNS environment. Kigerl et al. found that conditioned medium from CA macrophages was toxic to neurons, while conditioned medium from AA macrophages was not and could even induce axonal outgrowth across a gradient of inhibitory substrate [39]. Microglia activated by IL-4 are able to support oligodendrogenesis and some neurogenesis from adult neuronal progenitor cells, indicating a role in CNS repair for these microglia [40]. Furthermore, in microglia IL-4 induces the expression of genes typical for alternative activation and IL-4 deficiency in the CNS led to exacerbation of EAE [41].

The aim of the current study was to determine the functional characteristics of CA and AA macrophages, with respect to migration, motility, adhesion and phagocytosis, in the context of the CNS. We demonstrate for the first time that CA and AA macrophages behave differently under influence of the conditioned media of CNS cells. Furthermore, motility, adhesion and phagocytosis, cytoskeletal functions, varied between CA and AA macrophages. This was due to variation in cytoskeletal organization, activity of RhoA and Rac, and ROS production.

## Materials and methods

### Animals

For neuronal and astrocyte cultures, timed pregnant C57BL/6 mice were obtained from Charles River (Maastricht, The Netherlands). For isolation of bone marrow, adult C57BL/6 mice were used from Charles River.

All experiments were performed according to the guidelines of the local University Committee on Animal Welfare, which follow the European Communities Council Directive (86/609/EEC).

### Macrophages

Bone marrow derived macrophages were generated as described previously [42]. Bone marrow was flushed from femurs and tibias of C57/BL6 mice and cultured for 1 week in complete macrophage medium (Dulbecco modified Eagle's minimal essential medium (DMEM) (Invitrogen, Breda, the Netherlands) supplemented with 10% fetal calf serum (FCS) (Invitrogen), 15% conditioned medium from macrophage-colony stimulating factor-secreting L929 fibroblasts and 2% penicillin/streptomycin-glutamine (Lonza, Breda, the Netherlands) at 37°C. After 7-10 days in culture adherent cells were approximately 95% pure macrophages and cells were used for experiments.

The CA phenotype was induced by exposing macrophages for 48 hr to 5 × 10^3 ^U/ml IFN-γ (U-Cytech, the Netherlands) and 10 ng/ml *Escherichia coli *LPS (026:B6; Sigma-Aldrich, Zwijndrecht, the Netherlands) in the culture medium. AA macrophages were prepared by exposure to 10 ng/ml IL-4 (Invitrogen) in the culture medium [3,43]. Control macrophages were cultured for the same period in medium alone.

Macrophages were harvested by 15 min incubation at 37°C with lidocaine (4 mg/ml in PBS; Sigma-Aldrich). Macrophages were washed and centrifuged for 5 min at 170 g.

The phenotype of the differently activated macrophages was determined using a Griess assay and FACS analysis. Using the Griess assay, nitrite, the stable end product of NO, was measured. 100 μl supernatant of the differently activated macrophages was added to 100 μl Griess reagent (0.1% anphthylene diamine dihydrochloride, 1% sulfanilamide and 2.5% H3PO4). The OD540 was measured on a Benchmark microplate reader (Bio-Rad laboratories, Veenendaal, the Netherlands) and the concentration of nitrite present in the supernatant was determined by linear regression from a standard curve using known concentrations of sodium nitrite.

### FACS analysis

To determine the expression of several markers on differently activated macrophages a FACS analysis was performed. In brief, 1*10^5 ^macrophages were washed and incubated with the first antibody (see Table 1) for one h. Subsequently, macrophages were washed and exposed to the secondary fluorescently labeled antibody (see Table 1) for one h. The extent of marker expression was analysed using flow cytometry (FACSCalibur, Becton Dickinson, Erembodegem, Belgium) combined with Cellquest Pro software (Becton Dickinson). Omission of the primary antibody was included as negative control. The mean fluorescent intensity (MFI) of the macrophages was measured and data of three separate experiments were averaged. To obtain a better insight into the relative upregulation of the markers on the differently activated macrophages the results were expressed relative to control macrophages.

**Table 1:** Antibodies and dilutions used in FACS analysis of macrophages

Primary antibody	Subtype	Dilution	Secondary antibody	Dilution
**F4/80 **(Serotec, Oxford, UK)	IgG	1:500	Anti-rat Alexa 488 (Invitrogen)	1:400

**MAC-1 **(Serotec, Oxford, UK)	IgG	1:500	Anti-rat Alexa-488 (Invitrogen)	1:400

**MR **biotinylated (Biolegend, Uithoorn, the Netherlands)	IgG	1:100	Streptavidin-Alexa 488 (Invitrogen)	1:400

**LFA-1 **produced in house	IgG	1:250	Anti-rat Alexa 488 (Invitrogen)	1:400

**CD29 **(BD Pharmingen, Breda, the Netherlands)	IgG	1:100	Anti-rat Alexa 488 (Invitrogen)	1:400

**CD11a-FITC **(eBioscience, Malden, the Netherlands)	IgG	1:100		

### Conditioned media

Neuron conditioned medium (NCM) was derived from neurons of C57/BL6 primary mouse CNS. Embryonic day 19 mouse pups were sacrificed and the brain was isolated. For neuronal cultures, the cortex was isolated and incubated with trypsin containing 0.1 mg/ml DNAse for 15 min at 37°C. The cell suspension was extensively washed and the neurons were triturated to create a single cell suspension. Neurons were cultured in complete neurobasal medium, consisting of incomplete neurobasal medium with 1% glutamax, 2% B27 and 0.01% gentamycin (all obtained from Invitrogen), at a concentration of 1 × 10^5 ^cells/ml. Beta-tubulin (Covance, Uden, the Netherlands) staining was performed and cultures were found to be approximately 90% pure (data not shown, rest are predominantly astrocytes and some microglia). After 2 days in culture NCM was harvested.

For astrocyte conditioned medium (ACM), the forebrain cortex of mouse pups was isolated and single cell suspension was generated. Cells were cultured for 1 week in complete medium which consisted of DMEM with high glucose, supplemented with 1% glutamax, 10% FCS and 0.01% gentamycin (all obtained from Invitrogen). After 1 week, new medium was added to the culture. Cultures were characterized using glial fibrillary acidic protein (GFAP, Sigma-Aldrich) staining and were found to be approximately 95% pure (rest are neurons and microglia, data not shown). After 48 hr of culturing, ACM was harvested.

Oligodendrocyte conditioned medium (OCM) was harvested during oligodendrocyte development. Primary rat oligodendrocytes were cultured as described previously [44]. Briefly, oligodendrocyte precursors were cultured on poly-l-lysine coated cell culture plates for 2 days in a defined SATO medium [45] containing platelet derived growth factor-AA (PDGF-AA) and fibroblast growth factor-2 (FGF-2) in order to synchronize precursors to the bipolar oligodendrocyte-type II astrocyte (O2A) stage. Differentiation was induced by replacing the growth factors with 0.5% FCS in SATO medium. Medium was harvested from: (i) cells in the O2A stadium (designated as O2A medium); (ii) cells that had differentiated from O2A to the galactocerebroside (GC) stage (3 days differentiation, GC medium); (iii) cells that differentiated from GC to myelin basic protein (MBP) positive stage (7 days differentiation, MBP medium); finally from cells that had developed from MBP to MBP+ stage (10 days differentiation, designated MBP + medium).

### Fractioning of the conditioned media

In order to get insight into the range of molecular weight of the factors responsible for attraction of macrophages, conditioned media were fractioned based on molecular weight. Aliquots of the conditioned media were filtered using 10, 50 and 100 K Amicon Ultra centrifugal filter units as described by the manufacturer (Millipore, Amsterdam, the Netherlands). The aliquots were first filtered using the 10 kD filter, subsequently 50 and 100 kD, creating three fractions: one containing low molecular weight (< 10 kD), one with intermediate molecular weight (between 10 and 50 kD) and a fraction from 50 to 100 kD. The fractions were reconstituted in half of the original volume.

### Migration and motility

The migratory capacity of CA and AA macrophages was studied using a 48-wells micro chemotaxis chamber (Neuro Probe, Gaithersburg, USA) as described previously [46] with some modifications. In the bottom well, 25 μl of the conditioned media, control medium (neurobasal for NCM, DMEM for ACM and SATO medium for OCM), monocyte chemotactic peptide-1 (MCP-1) (20 ng/ml; Peprotech inc, London, UK) or formyl methionineleucyl-phenylalanine (fMLP) (10 nM; Sigma-Aldrich) was added. A filter with a pore size of 10 μm was used. In the upper chamber 2 *10^4 ^differently activated macrophages were added. The macrophages were left to migrate for 6 h. The side of the filter in direct contact with the upper chamber was washed and scraped clean of cells and the filter was subsequently stained using Coommassie Blue. The number of migrated cells was counted per 0.1 mm^2 ^using a scored eyepiece. From this the total number of migrated cells was calculated.

To determine motility, 5*10^4 ^CA and AA macrophages were seeded in culture plastic 96 wells plate (Greiner Bio One, Alphen a/d Rijn, the Netherlands) and cultured for 1 h. The 96-wells plate was placed in a time-lapse video microscope. Macrophages in each well were followed for 15 min and images were taken every 20 seconds at a 40 times magnification. The software program Track-It^® ^was used to calculate speed and distance moved as a measure for motility. All the cells present in a microscopic field were tracked manually.

### Adhesion

Macrophage adhesion to plastic and several extracellular matrix molecules (ECM) was determined as described previously [47]. CA and AA macrophages were harvested and labeled with 1 μM BCECF-AM (Invitrogen) for 15 min at 37°C. After labeling, cells were washed and 1 × 10^5 ^macrophages in 100 μl were seeded in the 96 wells plates. Macrophages were left to adhere for 2 h at 37°C and 5% CO_2_. After incubation, non-adherent cells were removed by washing 3 times in PBS and the remaining adherent cells were lysed with 0.1 N NaOH. Fluorescence was measured in a Fluostar24 (BMG labtechnologies, Offenburg, Germany). A standard curve with cell concentrations ranging from 5 × 10^3 ^to 1 × 10^6 ^cells/ml was made to determine the percentage of adhering cells.

To determine the adherence of differently activated macrophages to different ECM molecules, wells of a 96-well culture plate were coated with either collagen (type I from calf skin, Sigma-Aldrich), for 1 h at room temperature, or fibronectin (derived from human plasma; Roche, Almere, the Netherlands), for 1 h at 37°C.

We investigated the expression of adhesion receptors on differently activated macrophages using FACS analysis. We performed the FACS analysis as described above. The antibodies used are listed in Table 1.

### Actin cytoskeleton

The actin cytoskeleton of the differently activated macrophages was visualized using rhodamine phalloidin (Invitrogen). Macrophages were cultured on glass coverslips and stimulated in order to generate the different phenotypes. After 2 days, macrophages were fixed by incubation for 30 min with paraformaldehyde (4% in PBS). The macrophages were washed twice with PBS containing 0.1% Tween-20 (Sigma-Aldrich) and exposed to rhodamine phalloidin (1:300, Sigma-Aldrich) in PBS containing 0.1% Tween-20 for 1 h. To visualize nuclei, the cells were counterstained with Hoechst (Invitrogen) and afterwards embedded in mounting medium.

### RhoA and Rac activity G-LISA

RhoA and Rac activity in differently activated macrophages was determined using G-LISA activation assay kit (Cytoskeleton, Denver, USA) according to the manufacturer's description. In brief, macrophage cell lysate was added to a pre-coated 96-wells plate to which the active, GTP-bound form of the protein will bind. The bound protein was detected using a primary antibody to the protein and a secondary antibody linked to HRP. The signal was developed using OPD and absorbance was measured using a Benchmark microplate reader (Bio-Rad laboratories, Veenendaal, the Netherlands).

### Phagocytosis

The extent of phagocytosis of fluorescently labeled myelin and neuronal fragments by macrophages was determined using fluorescence activated cell sorter (FACS) analysis. Neuronal fragments were made by roughly pipetting 2 day old neuronal cultures, prepared as described previously [48], and labeling them with the lipophilic fluorescent dye 1,1',di-octadecyl-3,3,3'3'-tetramethylindocarbocyanine perchlorate (DiI) (Sigma-Aldrich). We adapted a protocol described previously for myelin phagocytosis^41^.

3*10^5 ^Macrophages were washed twice and incubated with fluorescently labeled myelin (10 or 20 μg/ml) for 1.5 h at 37°C. Macrophages were washed three times and harvested using 4 mg/ml lidocain (Sigma-Aldrich) for 15 min at 37°C. The macrophages were washed and analyzed using flow cytometry and Cellquest Pro software. Phagocytosis was displayed in percentages relative to control macrophages.

To determine the effect of CR3, small GTPases and ROS on phagocytosis, anti-CR3 antibody (10 μg/ml), quercetin (300 μM, Kaden Biochemicals, Hamburg, Germany), luteolin (300 μM, Kaden Biochemicals) and diphenylene iodonium (DPI) (Sigma-Aldrich) were added to macrophages during the phagocytosis.

To exclude that the phagocytosis measured on the FACS is only binding, confocal images were taken of macrophages after phagocytosis. Macrophages were cultured and stimulated for 48 h with either IFN-γ and LPS or IL-4 to generate the different phenotypes on cover slip glasses. The macrophages were incubated with DiI labeled myelin or neuronal fragments for 1.5 h at 37°C. Afterwards, the macrophages were stained using MAC-1 and the Alexa 488 labeled anti-rat secondary antibody. Images were taken on a Leica TCS SP2 confocal microscope.

### Statistical analysis

The data are expressed as mean of 3 to 4 separate experiments performed in duplo (± SEM). Statistics were performed in SPSS (15.0.0, Chicago, USA). The motility, migration, adhesion, G-LISA and phagocytosis experiments were analysed using one-way ANOVA with Bonferroni correction. A p-value of less than 0.05 was considered significant.

## Results

### Phenotype

**Figure 1:**
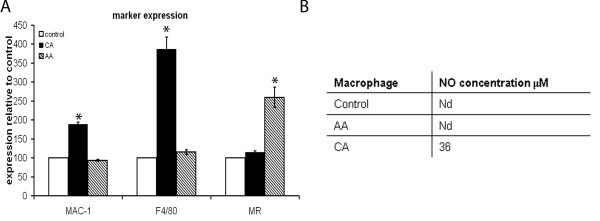
**Analysis of macrophage phenotype**. A) Using FACS analysis the expression of markers for the differently activated phenotype was determined. CA macrophages expressed significantly more MAC-1 and F4/80 compared to AA and control macrophages. MR expression was significantly higher on AA macrophages compared to control and CA macrophages. B) NO production was undetectable in both control and AA macrophages. CA macrophages did produce NO.

The phenotype of the differently activated macrophages was determined by investigating both marker expression and NO production. The expression of Mac-1, F4/80 and MR was determined using FACS analysis. MR is a longstanding marker for the AA phenotype [3]. Macrophages stimulated *in vitro *with IFN-γ and LPS have been found to express high levels of Mac-1 [49]. In accordance with literature, we observed a significant upregulation of the immunoreactivity for Mac-1 and F4/80 on CA macrophages, while the immunoreactivity for MR was increased on AA macrophages (Figure 1A).

CA macrophages are known to produce NO and ROS. Therefore, the production of NO was taken as a marker for the CA phenotype. Significant amounts of NO were produced by CA macrophages, while no detectable amounts of NO were produced by AA and control macrophages (Figure 1B).

### CA and AA macrophages migrate differently towards conditioned media from CNS cells

To investigate whether CA and AA macrophages migrate towards different CNS cell types, a 48-wells micro chemotaxis chamber was used. Macrophages migrated over a filter in response to conditioned medium harvested from neurons, astrocytes and several developmental stages of oligodendrocytes.

**Figure 2:**
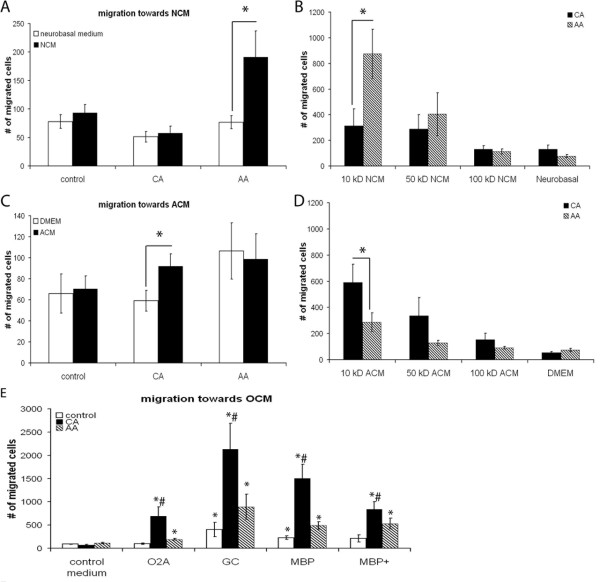
**Migration of macrophages towards conditioned medium from several CNS cell types**. Migration towards conditioned medium from various CNS cell types and chemokines by *in vitro *stimulated bone marrow derived macrophages was studied using a 48-wells micro chemotaxis chamber. The data are expressed as the mean of 4 separate experiments (n = 4) ± SEM. A) AA macrophages migrate in significantly higher numbers towards NCM compared to control medium, 191 and 77 respectively and also compared to CA macrophages, 57. * = p < 0.05. B) AA macrophages migrated in significantly higher numbers towards the fraction of the NCM containing proteins smaller than 10 kD compared to CA macrophages. Both the fractions containing proteins smaller than 10 kD and between 10 and 50 kD significantly enhanced migration of AA macrophages compared to control medium. * = p < 0.05. C) CA macrophages migrate in significantly higher numbers towards ACM compared to control medium, 92 and 59 macrophages respectively. AA macrophages did not specifically migrate towards ACM. No difference was seen in the number of migrated macrophages towards ACM between CA and AA macrophages. * = p < 0.05. D) The fraction of the ACM containing proteins smaller than 10 kD significantly enhanced the migration of CA macrophages compared to control medium and AA macrophages. * = p < 0.05. E) The different stages of oligodendrocyte development conditioned medium significantly enhanced migration of both CA and AA macrophages compared to oligodendrocyte control medium, however CA macrophages migrated in significantly higher numbers compared to AA and control macrophages. * = p < 0.05 is significantly different from control medium; # is p < 0.05 different from AA and control macrophages.

AA macrophages migrated in significantly higher numbers towards NCM compared to control neurobasal medium. CA macrophages were not attracted towards NCM (Figure 2A). In order to determine the molecular weight range of the factors that act chemotactic on AA macrophages, NCM was fractioned according to molecular weight. The NCM fraction containing proteins smaller than 10 kD was responsible for this effect (Figure 2B).

In contrast, migration of CA macrophages was significantly enhanced towards ACM compared to control medium (Figure 2C). The ACM fraction containing molecules smaller than 10 kD was responsible for the attraction of CA macrophages (Figure 2D). Fractions containing proteins larger than 10 kD did not significantly attract macrophages. AA macrophages did not migrate in higher numbers towards ACM compared to control medium.

The conditioned media of different stages of oligodendrocyte development, from precursors to mature oligodendrocytes, significantly attracted CA and AA macrophages compared to the control medium (Figure 2E). Control macrophages migrated in significantly higher numbers towards the GC and MBP developmental stages compared to control medium. Specifically the GC developmental stage induced enhanced migration in all macrophage phenotypes. CA macrophages were attracted in significantly higher numbers compared to both control and AA macrophages.

### AA macrophages have a higher motility than CA macrophages

**Figure 3:**
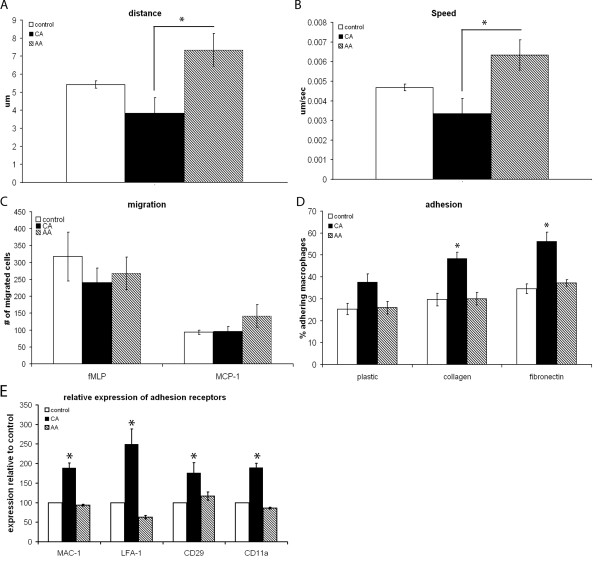
**Motility and migratory capacity**. Motility of *in vitro *stimulated bone marrow derived macrophages was determined by timelapse video microscopy. Speed and distance covered were determined using TrackIt software. The migration towards fMLP and MCP-1 was used as a measure for migratory capacity of in vitro stimulated bone marrow derived macrophages. Adhesion was determined by plating fluorescently labeled macrophages on (un)coated 96 wells plates, washing the plates after 2 h and measuring fluorescence. Data are the mean of 3 separate experiments (n = 3) ± SEM. *= p < 0.05. A) During the recorded time the average distance AA macrophages covered was significantly enhanced twofold compared to CA macrophages. The distance was recorded in μm. B) AA macrophages had a twofold higher speed compared to CA macrophages. Speed was recorded in μm/sec. C) No difference in migration was seen between the different macrophage phenotypes when comparing migration towards either fMLP or MCP-1. D) CA macrophages displayed a trend towards higher adherence on plastic compared to control and AA macrophages. On fibronectin and collagen adherence of CA macrophages was twofold higher compared to both control and AA macrophages. E) The expression of adhesion receptors was higher in CA macrophages compared to both AA and control macrophages.

Since we observed this striking effect on migration, we next determined the motility of CA and AA macrophages using a time-lapse video microscope. AA macrophages were significantly more motile compared to CA macrophages (Figure 3A and 3B). CA macrophages appeared to move very little.

Since motility was lower in CA macrophages compared to AA macrophages, we wanted to determine whether the intrinsic migratory capacity of CA macrophages was also reduced. Migration towards the potent macrophage attractors MCP-1 and fMLP, a synthetic analogue of a bacterial signal peptide, was used to address this question. As can be seen in Figure 3C, AA and CA macrophages migrated in comparable numbers towards fMLP and MCP-1, indicating that the intrinsic migratory capacity did not differ.

We next investigated whether the difference in motility was related to altered adherence. Therefore, the adhesion capacity of the differently activated macrophages to culture plastic and ECM molecules was measured. On culture plates CA macrophages displayed a trend towards increased adherence compared to control and AA macrophages (Figure 3D). After coating the culture plates with either collagen or fibronectin, the percentage of CA macrophages that adhered was enhanced twofold compared to both control and AA macrophages. We next investigated whether altered presence of adhesion receptors could explain the differences in adherence seen. We determined the expression of MAC-1, lymphocyte function associated antigen-1 (LFA-1), CD29 and CD11a. CA macrophages expressed significantly more adhesion receptors compared to both control and AA macrophages (Figure 3E).

### Actin cytoskeleton of CA and AA macrophages

**Figure 4:**
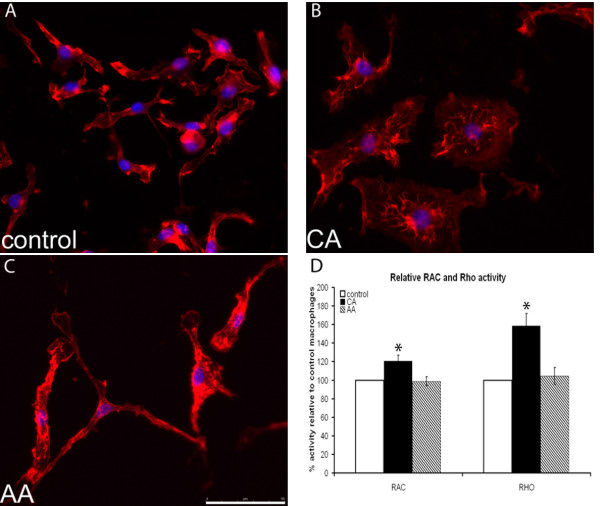
**Cytoskeleton of differently activated macrophages**. A-C) Visualization of actin cytoskeleton, red: actin (rhodamine phalloidin); blue: nuclear staining (Hoechst). Representative pictures are shown. CA macrophages appear flattened and rounded. AA macrophages are more stretched and elongated. Control macrophages appear to possess morphology in between the two extremes, with some cells being more elongated while others are more rounded. The scale bar is 50 μm and a 40 times magnification was used. D) Rac and Rho activity. In CA macrophages small GTPases are more activated compared to both control and AA macrophages.

Migration, motility and adhesion are functions that are all linked to the cytoskeletal organization of cells. Therefore we determined whether differences in the cytoskeleton could be observed between the macrophage phenotypes, by staining both phenotyopes for F-actin (Figure 4A-C). Remarkably, the morphology of CA macrophages appeared more rounded with an enhanced number of lamellipodia compared to control macrophages. In contrast, AA macrophages were elongated compared to control cells. In CA macrophages the staining pattern showed clustering of F-actin around the nucleus, whereas in AA macrophages the staining was more prominent at the border of the cell, indicating possible focal adhesions.

Next we determined the activity of small GTPases RhoA and Ras in activated macrophages, since they are involved in actin cytoskeleton modulation. The activity of both RhoA and Rac was significantly higher in CA macrophages compared to both control and AA macrophages (Figure 4D).

### Phagocytosis of myelin is higher in CA compared to AA macrophages

Phagocytosis, a crucial macrophage function, is also linked to the activity of small GTPases and cytoskeletal reorganisation. We determined the myelin phagocytosis capacity of the differently activated macrophages.

**Figure 5:**
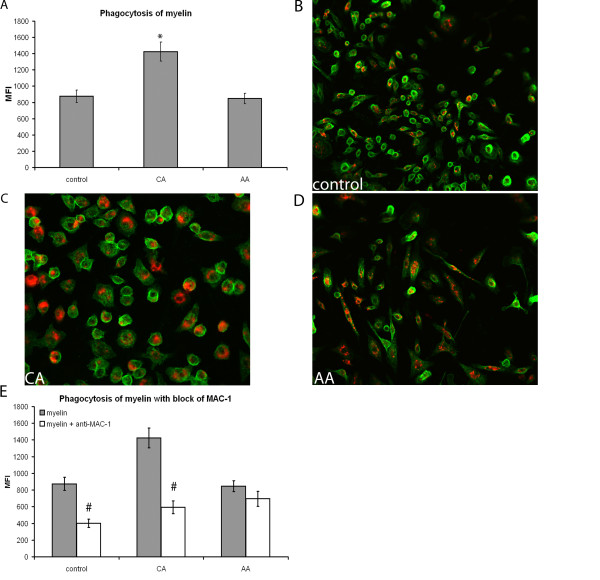
**Phagocytosis of myelin by differently activated macrophages**. Phagocytosis of fluorescently labeled myelin by activated bone marrow derived macrophages and expression of CR3 was determined using flow cytometry. MFI was measured and data are the mean of 4 separate experiments (n = 4) ± SEM. *= p < 0.05 and #= p < 0.05. A) CA macrophages phagocytosed significantly more myelin compared to control and AA macrophages. B-D) Visualization of myelin internalization by phagocytosis, red: DiI labeled myelin; green: Mac-1 on macrophage membrane. Representative images, taken on a confocal microscope, are shown. Red myelin is seen inside the green labeled membrane of the differently activated macrophages. All macrophage phenotypes appear to have taken up myelin. A 20 times magnification was used. E) Using anti-MAC-1 antibody, phagocytosis of myelin by CA and control macrophages could be blocked significantly, as indicated by #. Anti-MAC-1 antibody did not affect myelin phagocytosis by AA macrophages.

CA macrophages had a significant twofold increase in the phagocytosis of myelin compared to both control and AA macrophages (Figure 5A). In order to ensure that phagocytosis had taken place, we showed internalization of myelin by confocal microscopy (Figure 5 B-D). DiI labeled myelin was visible within the green labeled membranes of macrophages, showing that phagocytosis of myelin had indeed taken place. Since Mac-1, also called CR3, has been shown to be involved in myelin phagocytosis [50] and we observed a significantly higher expression of Mac-1 by CA macrophages compared to both control and AA macrophages (Figure 1A and 3E) we determined whether phagocytosis of myelin could be blocked using anti-CR3 antibodies. Myelin phagocytosis could be blocked using anti-CR3 antibodies in CA macrophages and control macrophages. The block of myelin phagocytosis by anti-CR3 antibodies in AA macrophages was not significant (Figure 5B).

**Figure 6:**
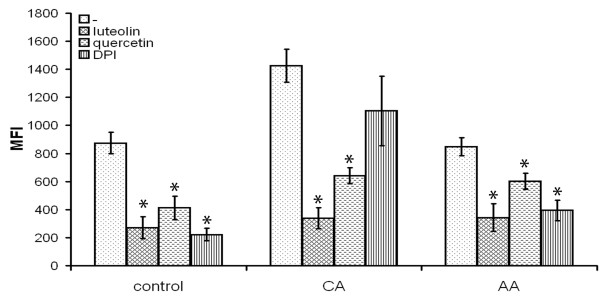
**Influence of DPI and flavonoids on phagocytosis of myelin**. To determine the influence of DPI, a NADPH oxidase blocker, and flavonoids on myelin phagocytosis, these substances were added together with fluorescently labeled myelin. Phagocytosis is determined as MFI of the macrophages. Data are the mean of 4 separate experiments (n = 4) ± SEM. *= p < 0.05. Both luteolin and quercetin significantly blocked phagocytosis of myelin by control, CA and AA macrophages. DPI only significantly reduced phagocytosis of myelin by control and AA macrophages. DPI did not significantly decrease phagocytosis of myelin by CA macrophages.

Next to CR3, ROS have also been implicated in the induction of phagocytosis. We therefore tested the effect a specific NADPH oxidase inhibitor, diphenylene iodonium (DPI), on myelin phagocytosis. Blocking ROS production, by DPI, reduced myelin phagocytosis by control and AA macrophages significantly (Figure 6). However, exposure to DPI did not have a significant effect on myelin phagocytosis by CA macrophages (Figure 6). Finally, flavonoids were used to block phagocytosis, since flavonoids are able to influence phagocytosis at multiple levels, such as ROS production and cytoskeleton mobilization. Using quercetin and luteolin, phagocytosis of myelin by CA, AA and control macrophages was blocked (Figure 6).

### Phagocytosis of neuronal fragments is higher in CA compared to AA macrophages

During neuroinflammatory diseases not only myelin debris is present, but also neuronal debris. Phagocytosis of myelin is well investigated, which is not the case for neuronal debris. Indications that neuronal debris is phagocytosed, comes from MS research. Macrophages were suggested to transport neuronal debris outside the CNS, since macrophages in cervical lymph nodes of MS patients contained neuronal antigens [51]. Therefore, we investigated the phagocytosis of neuronal fragments, obtained by roughly pipetting a monolayer of neurons as a model for neuronal debris, by differently activated macrophages.

**Figure 7:**
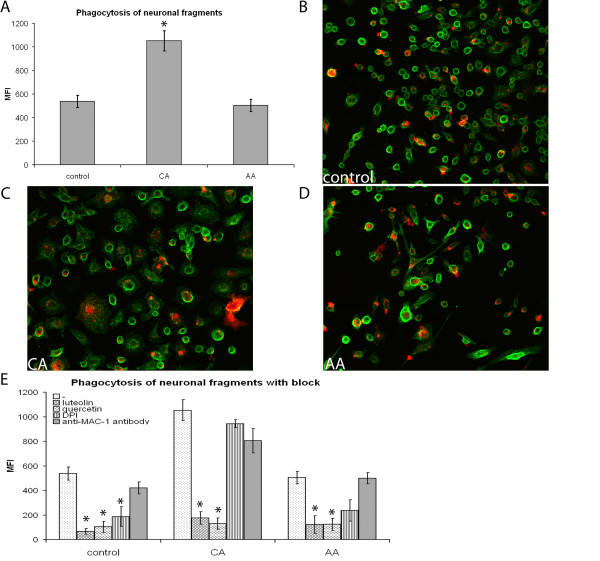
**Phagocytosis of neuronal fragments by differently activated macrophages**. Phagocytosis of fluorescently labeled neuronal fragments by activated bone marrow derived macrophages was determined using flow cytometry. Phagocytosis was blocked using anti-MAC-1 antibodies, luteolin, quercetin and DPI. Data are the mean of 4 separate experiments (n = 4) ± SEM. *= p < 0.05 and #= p < 0.05. A) CA macrophages phagocytosed significantly more neuronal fragments compared to control and AA macrophages. B-D) Visualization of neuronal fragment internalization by phagocytosis, red: DiI labeled neuronal fragments; green: Mac-1 on macrophage membrane. Representative images, taken on a confocal microscope (Leica TCS SP2), are shown. Red labelled neuronal fragments are clearly present within the green labelled membrane of the differently activated macrophages. Some binding is also present, with the neuronal fragments being present outside the membrane. A 20 times magnification was used. E) To determine whether phagocytosis could be blocked macrophages were exposed to luteolin, quercetin, MAC-1 (anti-CR3 antibody) or DPI (NADPH oxidase blocker). Phagocytosis of neuronal fragments by all types of macrophages was significantly reduced by exposure to luteolin and quercetin. Using MAC-1 antibody did not reduce neuronal fragment phagocytosis. DPI led to a significant reduction in control macrophages, however, CA and AA macrophages did not reduce phagocytosis significantly.

Phagocytosis of neuronal fragments was significantly enhanced by CA macrophages compared to both control and AA macrophages (Figure 7A). The phagocytosis of neuronal fragments did not differ between control and AA macrophages. To ensure that phagocytosis had taken place, we investigated internalization of neuronal fragments by confocal microscopy (Figure 7 B-D). All macrophage phenotypes were able to phagocytose neuronal fragments, since red labeled neuronal fragments were present within green labeled membranes. Some binding of neuronal fragments outside the macrophage membrane was also visible. The phagocytosis of neuronal fragments could be significantly blocked using luteolin and quercetin in all macrophage subtypes (Figure 7E). MAC-1 did not reduce phagocytosis of neuronal fragments. DPI only reduced phagocytosis in control macrophages.

## Discussion

In this study we focused on functional consequences of differential activation of macrophages for cytoskeleton-associated functions as migration, motility, adhesion and phagocytosis in the CNS. These properties may have considerable impact on the local effects of macrophage subtypes during neuroinflammation. We observe that different CNS cell types have diverging effects on the migration of CA and AA macrophages. Furthermore we show that activation status affects motility, adhesion and phagocytosis of differently activated macrophages. Finally, the actin cytoskeleton appeared differently organized between CA and AA macrophages.

This study shows that AA and CA macrophages differ in the CNS cell types towards which they migrate. AA macrophages were significantly attracted towards NCM. Especially small proteins, < 10 kD, were responsible for the preferential attraction of CA versus AA macrophages, suggesting that chemokines could be responsible for this effect. Interestingly, human AA macrophages have increased expression levels of CXCR4, the receptor for CXCL12 which is expressed in cultured neurons [52] and in normal brain [53], while CA macrophages have higher expression levels of CCR7 [54]. Furthermore, the migration towards the < 10 kD fraction of the medium induced a higher migration in AA macrophages compared to the whole medium, indicating that molecules present in the higher molecular weight fractions could be inhibiting migration. We observed that murine CA macrophages were significantly attracted towards ACM and oligodendrocyte conditioned medium. Using ACM, small proteins, < 10 kD, in the conditioned media attracted macrophages, suggesting that astrocytes release cytokines or chemokines. Again the lower molecular weight fraction of the medium induced a higher migration in CA macrophages compared to the complete medium, indicating the presence of inhibitory molecules in the whole medium. Finally, OCM, specifically of the GC developmental stage, attracted macrophages to a much higher extent compared to both NCM and ACM. The cells in the GC developmental stage are differentiating oligodendrocyte precursors. *In vivo*, macrophages are found both in areas of demyelination as well as in areas of remyelination. Furthermore, remyelination correlated with the number of oligodendrocytes and macrophages [55,56]. The fact that the OCM was derived from rat oligodendrocytes and the macrophages used for migration were murine could also play a role in the migration. Since in humans little research has been performed on differently activated macrophages within the CNS, no clinical data is available on the preferential association of differently activated macrophages with specific CNS cell types in human pathology.

Our results showed that the differences in migration were not due to intrinsic differences in migratory capacity, since fMLP and MCP-1 attracted both types of macrophages in comparable levels. Both fMLP and MCP-1 are potent chemoattractants [57,58]. Subtle differences in chemoattraction, due to differences in chemokine receptor expression, might be masked due to the high potency of these molecules.

One caveat in our study is that we used the supernatant of resting cultures to determine chemoattraction, which does not resemble the neuroinflammatory CNS. The cells in the inflamed CNS might secrete factors that were not present in our study, possibly changing the chemoattraction of the CA and AA macrophages. During EAE and MS, MCP-1 expression is upregulated in the CNS and in EAE a close association was observed between increased expression of MCP-1 and relapses [59]. MCP-1 expression is mainly found in reactive astrocytes and macrophages [60,61]. Since MCP-1 attracted all subtypes of macrophages equally, this would indicate that in MS lesions all macrophage subtypes would be able to migrate equally towards these reactive astrocytes, since no differences were observed in migration towards MCP-1.

Although the intrinsic migratory capacity, determined using fMLP and MCP-1, did not differ between CA and AA macrophages, we observed significantly higher spontaneous motility in AA macrophages compared to CA macrophages. An important factor for motility is the adhesive capacity of macrophages. Adhesion can also be influenced by the cytoskeletal organization [62,63]. We showed that CA macrophages adhered to a higher extent to culture plates and ECM molecules compared to AA macrophages, confirming that adhesion might also have an impact on the increased motility and migration towards NCM of AA macrophages. Our findings were in line with a previous study, observing that glucocorticoid stimulated murine monocytes, which display some features of AA macrophages, adhered less and were more motile than control cells [64]. We observed enhanced expression adhesion receptors in CA macrophages. Lymphocyte function associated antigen-1 (LFA-1), MAC1 (CR3), CD29 and CD11a expression was higher in CA macrophages compared to AA macrophages. The increased expression of adhesion receptors in macrophages stimulated with IFN-γ and LPS is in line with previous studies [49,65] and with our current finding that CA macrophages adhere better when compared to AA macrophages.

We observed that AA macrophages were more elongated, whereas CA macrophages were rounded. This is in line with findings in human M1 and M2 macrophages. Human M2 macrophages, analogous to AA macrophages, appeared stretched with a spindle-like morphology, while human M1, analogous to CA macrophages, macrophages appeared rounder with a "fried-egg" morphology [66]. The cytoskeletal organization differed between AA and CA macrophages, in that we found clusters of actin around the nucleus in CA macrophages, while the actin cytoskeleton of AA macrophages was more prominent at the border of the cell. Rho and Rac activity was higher in CA macrophages compared to both control and AA macrophages, possibly underlying the differences seen in cytoskeletal organization. These results indicate that during the transition to the AA phenotype macrophages become increasingly stretched and motile allowing migration into tissues, while transition to the CA phenotype leads to decreased motility and a round morphology, probably limiting migration.

Phagocytosis of myelin was higher in CA versus AA and control macrophages. We wanted to determine the mechanism behind this difference. CR3 is implicated in myelin phagocytosis [67]. We found a significantly higher expression of CR3, also called MAC-1, on CA macrophages compared to AA macrophages. This is in line with previous data showing upregulation of CR3 after IFN-γ and LPS treatment of macrophages [49]. Myelin phagocytosis by CA macrophages could be blocked using anti-CR3 antibodies. A slight reduction in myelin phagocytosis was seen after exposure of AA macrophages to anti-CR3 antibodies. These results indicate that CR3 plays an important role in myelin phagocytosis by both control and CA macrophages, while in AA macrophages another mechanism of phagocytosis is dominant. Next to the phagocytosis of myelin, the CA macrophages also phagocytosed significantly more neuronal fragments compared to control and AA macrophages. CR3 is not involved in the phagocytosis of neuronal fragments, since addition of anti-CR3 antibodies did not reduce phagocytosis by any of the macrophage subtypes.

In rat macrophages myelin phagocytosis was ROS dependent [68]. Blocking ROS production by treatment with DPI blocked phagocytosis of myelin by AA and control macrophages, suggesting that ROS play an important role in myelin phagocytosis in these macrophages. The effect on CA macrophages was not significant, indicating that ROS do not play a significant role here. This could be due to the fact that myelin phagocytosis is CR-3 mediated in CA macrophages, while this does not seem to play a major role in either AA or control macrophages. Phagocytosis of neuronal fragments was reduced by DPI only in control macrophages, indicating an important role for ROS in control macrophage phagocytosis. Both CA and AA macrophages did not reduce phagocytosis of neuronal fragments after exposure to DPI, suggesting a specific mode of uptake similar to myelin uptake by CA macrophages via CR3.

The flavonoids quercetin and luteolin significantly reduced macrophage phagocytosis of both myelin and neuronal fragments by all types of macrophages. Previously, myelin phagocytosis was blocked after exposure to quercetin and luteolin in a macrophage cell line [69]. Quercetin and luteolin have anti-oxidant properties [69], suggesting that scavenging of ROS is an important factor in phagocytosis. Furthermore, they inhibit nuclear factor-kappa B, thereby inhibiting pro-inflammatory cytokine secretion and NO production [70,71]. However, flavonoids do more than scavenge. Quercetin could also mediate its effects due to the inhibition of protein tyrosine kinases, which are involved in the signaling of the engulfment phase of CR3 mediated phagocytosis [72]. Luteolin has effects on the actin cytoskeleton [73,74] through Rho and Rac [75] and could thereby affect phagocytosis.

In conclusion, the neuronal damage that occurs during neuroinflammatory diseases, such as MS, seems to be correlated to clinical disability [27,76]. CA macrophages have a lower ability to migrate, since they adhere strongly to the ECM and are generally less motile, limiting the amount of bystander damage due to ROS and NO secreted by the CA macrophages. Altogether this would contribute to a limited lesion expansion. Due to the fact that AA macrophages are more motile, adhere less to the ECM and are attracted by NCM, they could migrate towards neurons and locally release growth factors where they are most needed. AA macrophages are considered to be growth promoting and can secrete neurotrophic factors [30]. Therefore, skewing macrophages towards an AA phenotype could be a novel avenue for the development of new therapeutic strategies in neuroinflammatory diseases. Several studies have found therapeutic effects of AA macrophages. *In vitro *generated AA macrophages reduced renal and pancreatic injury in a model for murine diabetes [77]. In a model for colitis the injection of AA macrophages ameliorated the disease [78]. In spinal cord injury addition of multipotent adult progenitor cells reduced axonal dieback induced by macrophages [79]. *In vitro *these multipotent adult progenitor cells induced an AA phenotype in macrophages [79]. Treatment with anti-CCL22 [80] and 2-arachidonoylglycerol [81], which were found to skew macrophages to an alternatively activated phenotype, increased the presence of AA macrophages in the lesions and ameliorated EAE disease course. Administration of AA macrophages reduced the development of relapses during the relapsing EAE model [82]. Adoptive transfer of AA macrophages induced using glatiramer acetate reversed clinical EAE [83]. Finally, in MS patients a deficiency in negative regulation of macrophage activation by Src homology region 2 domain-containing phosphatase-1 (SHP-1) was observed, leading to an enhanced response of these macrophages to both LPS and IL-4 [84,85]. This indicates that lesion environment determines the activation status of macrophages. Similar results have been found in a mouse model of Alzheimer's disease, since mRNA levels for markers of the CA and AA phenotype were observed to be upregulated in microglial cells, indicating that these microglia express functional characteristics of both CA and AA macrophages [86]. Together with the data from our current study, this supports the hypothesis that the "setting" of the innate immune system is crucial for disease outcome in inflammatory diseases of the CNS such as MS and SCI.

## List of abbreviations

AA: alternatively activated; ACM: astrocyte conditioned medium; CA: classically activated; CNS: central nervous system; CR3: complement receptor 3; DMEM: Dulbecco modified Eagle's minimal essential medium; DiI: 1,1',di-octadecyl-3,3,3'3'-tetramethylindocarbocyanine perchlorate; DPI: diphenylene iodonium; EAE: experimental autoimmune encephalomyelitis; ECM: extracellular matrix; FCS: foetal calf serum; fMLP: formyl methionineleucyl-phenylalanine; GAP-43: growth associated protein 43; GFAP: glial fibrillary acidic protein; IFN-γ: interferon-gamma; IL: interleukin; iNOS: inducible nitric oxide synthetase; IR: immunoreactivity; LFA-1: lymphocyte function associated antigen-1; LPS: lipopolysaccharide; MBP: myelin basic protein; MCP-1: monocyte chemotactic peptide-1; MMP: matrix metalloproteinase; MR: mannose receptor; MS: multiple sclerosis; NADPH-oxidase: nicotinamide adenine dinucleotide phosphate-oxidase; NCM: neuronally conditioned medium; NF: neurofilament; NO: nitric oxide; OPC: oligodendrocyte precursor cell; PBS: phosphate buffered saline; ROS: reactive oxygen species; SCI: spinal cord injury

## Competing interests

The authors declare that they have no competing interests.

## Authors' contributions

EV was involved in data acquisition, data analysis and statistical analysis. EV drafted the manuscript. DH performed the phagocytosis and FACS analysis assays. WB contributed the oligodendrocyte conditioned medium and critically reviewed the manuscript. HdV participated in the data analysis and conceptualization. CD and CT participated in study design, conceptualization, data analysis and helped to draft the manuscript. All authors have read and approved the final version of the manuscript.
